# Research progress of multimodal biomarkers in the early diagnosis of mild cognitive impairment in Parkinson’s disease

**DOI:** 10.3389/fneur.2025.1652378

**Published:** 2025-08-29

**Authors:** Huiping Guo, Xiaoming Shen, Mengke Lyu, Guangsheng Zhou, Chongchong Chen, Baofu Xing, Yanming Xie

**Affiliations:** ^1^Department of Encephalopathy, The First Affiliated Hospital of Henan University of Traditional Chinese Medicine, Zhengzhou, China; ^2^The First Clinical Medical College, Henan University of Chinese Medicine, Zhengzhou, China; ^3^Institute of Basic Research in Clinical Medicine, China Academy of Chinese Medical Sciences, Beijing, China

**Keywords:** Parkinson’s disease-mild cognitive impairment, multimodal biomarkers, early diagnosis, machine learning algorithm, review

## Abstract

Parkinson’s disease (PD), as a common neurodegenerative disorder, its associated mild cognitive impairment (PD-mild cognitive impairment, PD-MCI) is a key prodromal stage in the progression to PD-dementia (PDD). Cognitive dysfunction seriously affects the quality of life of patients. Early diagnosis of PD-MCI is crucial for the prognosis of the disease. At present, traditional clinical diagnosis mainly relies on neuropsychological tests, but it has limitations such as low sensitivity and being easily interfered by subjective factors. It is difficult to achieve early and accurate identification of PD-MCI, which greatly affects the intervention timing and prognosis of the disease. This article summarizes the research progress of multimodal biomarkers in the early diagnosis of PD-MCI, mainly including the comprehensive application of neuroimaging biomarkers, humoral biomarkers, genetic and molecular biomarkers, digital biomarkers, and clinical assessment, providing new theoretical basis and technical paths for promoting the early diagnosis of PD-MCI. It is of great clinical significance and social value to assist in the formulation of individualized intervention strategies, delay the progression of diseases to dementia, improve the quality of life of patients, and reduce the medical burden on families and society.

## 1 Introduction

**Figure 1:**
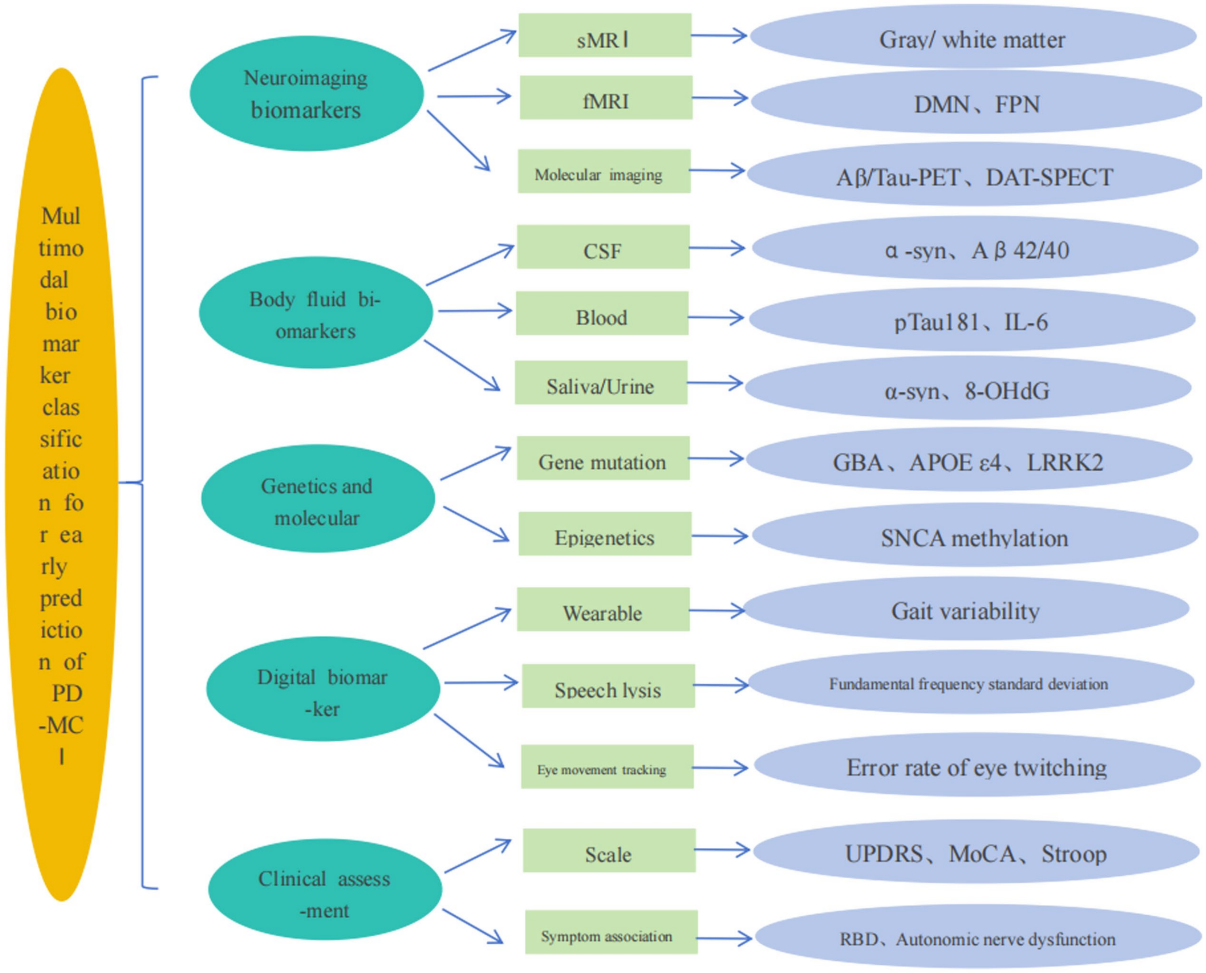
Classification and techniques of multimodal biomarkers.

Mild cognitive impairment (PD-mild cognitive impairment, PD-MCI) in Parkinson’s disease is the prodromal stage of PD-dementia (PDD), and its diagnosis and treatment are crucial for improving the prognosis of patients. Relevant epidemiological research shows that over 80% of PD patients have cognitive function changes, and approximately 30% of them will eventually develop into PDD (1). Between 20 and 33% of patients have PD-MCI at the time of diagnosis of PD, and as many as 57% of PD patients develop PD-MCI successively within 5 years after the first diagnosis (2, 3). In recent years, with the rapid development of big data, artificial intelligence and machine learning technologies, the application of multimodal biomarkers has increasingly gained favor in clinical research, providing powerful tools for the early diagnosis and prognosis of diseases. By aggregating and integrating multi-dimensional data such as neuroimaging, humoral biomarkers, genetics, digital phenotypes, and clinical assessment tools, the pathophysiological changes of PD-MCI can be reflected from different perspectives, providing support for conducting high-quality clinical research and precise diagnosis and treatment. The realization of precise identification of risk factors for PD-MCI, early intervention of the disease and improvement of prognosis provides evidence-based medical evidence, which is conducive to the formulation of effective prevention and treatment strategies in clinical practice. This article reviews the application of multimodal biomarkers in the diagnosis of PD-MCI, explores their different roles in clinical diagnosis, and looks forward to their potential value in the assessment of PD-MCI, aiming to provide a more comprehensive diagnostic strategy for clinical practice and offer references for future research directions (Figure 1).

## 2 Neuroimaging biomarkers

Imaging plays a crucial role in the early diagnosis of PD-MCI (Parkinson’s Disease with Mild Cognitive Impairment). Through imaging techniques such as Structural Magnetic Resonance Imaging (sMRI), Functional Magnetic Resonance Imaging (fMRI), Single Photon Emission Computed Tomography (SPECT), and Positron Emission Tomography (PET), researchers can identify structural and functional changes associated with PD-MCI. For instance, MRI can reveal atrophy in brain regions related to motor control, while PET is used to detect changes in dopamine transporter activity in the brain. These imaging biomarkers are not only helpful for early diagnosis but also provide key evidence for monitoring disease progression. Studies have shown that imaging-related indicators, such as changes in the basal ganglia, are closely correlated with the severity of clinical symptoms, making imaging an important tool for assessing PD-MCI patients (4).

### 2.1 Structural magnetic resonance imaging

Structural Magnetic Resonance Imaging (sMRI), utilizing surface-based morphometry (SBM) techniques, quantifies parameters such as cortical thickness, surface area, volume, and curvature, revealing the characteristic gray matter atrophy and white matter damage in PD-MCI patients. Changes in these parameters are closely related to dysfunction in specific cognitive domains, as reflected by abnormal striatal volume alterations in PD-MCI patients. A multicenter study found that cortical thickness in the frontal lobe (dorsolateral prefrontal cortex, DLPFC) and parietal lobe (inferior parietal cortex) of PD-MCI patients was significantly reduced compared to healthy controls, with the degree of atrophy positively correlating with declines in executive function (e.g., Stroop test) and working memory scores (5). A longitudinal cohort study showed that volume reduction in the temporal lobe (parahippocampal gyrus, entorhinal cortex) independently predicts episodic memory impairment in PD-MCI patients, with an atrophy rate 1.5 times faster than in PD patients without cognitive impairment (6). Compared to early PD patients without MCI, the PD-MCI group exhibited more pronounced white matter microstructural abnormalities. sMRI evidence indicated widespread reductions in white matter fiber tract integrity, along with significantly increased volumes of white matter hyperintensity lesions in periventricular and deep brain regions. Moreover, expansion of white matter hyperintensities in the left frontal and temporal lobes was significantly associated with worsening performance on free recall tests, suggesting that white matter damage in specific brain regions may underlie PD-MCI pathology (7–10).

### 2.2 Functional magnetic resonance imaging

Resting-state fMRI studies have shown (11) that PD-MCI patients exhibit significant weakening in the functional connectivity (FC) of the default mode network (DMN) between the hippocampus-inferior frontal gyrus, posterior cingulate cortex-posterior parietal lobe, anterior temporal lobe-inferior frontal gyrus, and middle frontal gyrus-middle temporal gyrus. Compared to controls, PD-MCI patients show markedly reduced bilateral prefrontal cortex intra-FC, characterized by decreased connectivity strength between the caudate nucleus and precuneus (12). Besides widespread FC reduction, studies have reported abnormal increases in FC between the midbrain-posterior cingulate cortex and left hippocampus-right cerebellar hemisphere in PD-MCI patients (13, 14). Apart from FC, local consistency (ReHo) values within the DMN positively correlate with cognitive function in PD and continuously decline with disease progression (15). The dynamic FC changes in the DMN, frontoparietal network (FPN), and salience network are more sensitive than structural imaging, with continuous alterations in the salience network showing the strongest association with PD cognitive impairment (PD-CI). Multiple task-based fMRI studies have found (16, 17) that during working memory tasks, PD-MCI patients exhibit significantly reduced activation in bilateral caudate nuclei, right putamen, and dorsolateral/ventrolateral prefrontal cortex, indicating dopaminergic-cortical circuit dysfunction. Additionally, cerebellar functional activity is generally weakened, suggesting damage to cerebello-cortical pathways or their involvement in cognitive regulation.

### 2.3 Dopaminergic system imaging

Dopamine transporter (DAT) single-photon emission computed tomography (SPECT) demonstrates early dopaminergic dysfunction and its association with cognitive impairment in PD-MCI patients by detecting dopaminergic nerve terminal density in the striatum. A multicenter study showed that DAT binding in the caudate and posterior putamen of PD-MCI patients was 30–40% lower than that of healthy controls, with caudate DAT loss significantly correlated with executive functions (such as Stroop test) and working memory (such as digit span test) scores (18). The Bäckman research team, through longitudinal DAT-SPECT tracking, found that PD patients with an annual striatal DAT binding decline rate exceeding 5% had a 3.2-fold increased risk of progression to MCI within 3 years, indicating that DAT loss could serve as an early predictive biomarker of cognitive decline (19). Serotonin (5-HT) positron emission tomography (PET) utilizes tracers (e.g., [11C] DASB, [18F]altanserin) to visualize serotonin transporter (SERT) or receptor distribution, revealing abnormalities in the raphe 5-HT system and its association with attention and emotional regulation deficits in PD-MCI. Studies show that compared to healthy controls, PD-MCI patients exhibit a 45% decrease in raphe SERT binding, which is positively correlated with Conners’ Continuous Performance Test (CPT) scores. Reduced 5-HT projections from the raphe to the prefrontal cortex may impair attentional control (20). Smith et al.’s longitudinal 5-HT PET study indicated that PD patients with an annual raphe SERT binding decline exceeding 8% have a 2.5-fold increased risk of developing MCI within 2 years (21). The Ballanger team, using the [18F] MPPF tracer, demonstrated elevated 5-HT1A receptor binding in the anterior cingulate cortex of PD-MCI patients, which is associated with depressive symptoms (e.g., BDI scores) and anxiety, suggesting that compensatory receptor upregulation might exacerbate mood disorders (22).

### 2.4 Metabolism and molecular imaging

Fluorodeoxyglucose positron emission tomography (FDG-PET) reveals the distinctive metabolic patterns of PD-MCI (Parkinson’s Disease Mild Cognitive Impairment) patients by measuring cerebral glucose metabolism rate, which is associated with impairments in specific cognitive domains. Huang et al.’s study demonstrated significantly reduced glucose metabolism in the prefrontal cortex (especially the dorsolateral prefrontal cortex, DLPFC) and the inferior parietal lobule in PD-MCI patients, with the degree of metabolic decline positively correlating with executive function scores (e.g., Stroop test, Digit Symbol Substitution Test) (23). Tau positron emission tomography (Tau-PET), employing specific tracers such as [18F]AV-1451 and [18F]MK-6240, visualizes tau protein deposits, illustrating the spatial characteristics of tau pathology in PD-MCI and their relationship to cognitive decline. Schöll et al. found that tau deposition in the midbrain positively correlates with dopaminergic neuron loss assessed by DaT-SPECT, suggesting the involvement of tau pathology in degeneration of the nigrostriatal pathway (24). Ossenkoppele et al. proposed that tau deposition in PD-MCI primarily occurs in the midbrain and limbic system, whereas in Alzheimer’s disease (AD) it is predominantly distributed in the temporoparietal lobes and neocortex, supporting specificity in pathological distribution (25).

## 3 Fluid biomarkers

Fluid biomarkers are crucial for the early identification of PD-MCI. Specific biochemical substances detected in blood and cerebrospinal fluid, such as neuron-specific enolase (NSE) and neurofilament light chain (NfL), are considered closely related to the degree of neuronal damage and stages of disease progression. The detection of these biochemical markers not only aids in identifying early PD-MCI patients but also serves to monitor disease progression and evaluate therapeutic efficacy (26). Due to their diversity and sensitivity, biochemical biomarkers hold broad application potential in clinical practice. Research on biomarkers continues to advance, and novel biomarkers, such as changes in α -synuclein and tau protein, have been confirmed to be associated with PD-MCI, offering new avenues for early clinical diagnosis (27).

### 3.1 Cerebrospinal fluid

In patients with PD-MCI, changes in cerebrospinal fluid (CSF) α -synuclein levels reflect the association between pathological progression and cognitive impairment. Numerous studies have shown that total α -synuclein levels in the CSF of PD-MCI patients are significantly lower than those in healthy controls and PD patients without cognitive impairment. Kang et al. found that decreased CSF α -synuclein levels in PD-MCI patients are related to midbrain atrophy and weakened functional connectivity of the default mode network (DMN), suggesting it may serve as a surrogate marker for neuronal loss (28). Parnetti et al.’s review indicates that reduced CSF α -synuclein is associated with degenerative changes in the frontostriatal pathway in PD-MCI patients, possibly affecting executive function through synaptic dysfunction (29). Unlike total α -synuclein, its oligomeric form (oligomeric α -syn) is significantly elevated in PD-MCI patients and correlates with cognitive deterioration. Shahnawaz et al. developed a novel detection method and found that CSF oligomeric α -syn levels in PD-MCI patients are three times higher than those in healthy controls and negatively correlated with Montreal Cognitive Assessment (MoCA) scores (30). Abnormal phosphorylation of Tau protein (such as p-tau181, p-tau231) is a typical marker of Alzheimer’s disease (AD), and elevated CSF Tau levels are also observed in PD-MCI patients; however, its pathological distribution and clinical significance differ from those in AD-MCI. Alves et al. found that CSF p-tau181 levels in PD-MCI patients are significantly higher than in PD patients without cognitive impairment, but the increase is less pronounced compared to AD-MCI patients and lacks strong correlation with hippocampal atrophy, suggesting that Tau pathology in PD-MCI may be confined to subcortical regions (such as the brainstem and limbic system) (31). Hall et al. demonstrated through proteomic analysis that the Tau phosphorylation patterns (such as p-tau231) in PD-MCI patients differ from those in AD-MCI and may contribute to synaptic toxicity formation together with α -synuclein (32).

### 3.2 Blood

The levels of α-synuclein and synaptic proteins such as GAP43 and SNAP25 in blood exosomes change and can serve as non-invasive biomarkers. Abnormal oligomers of α-synuclein can spread between neurons via exosomes, accelerating pathological propagation. Stuendl et al. observed a significant increase of α-synuclein oligomers in the blood exosomes of PD-MCI patients, which positively correlated with oligomer concentrations in cerebrospinal fluid (CSF), indicating it can replace CSF testing (33). Shi et al. used single molecule array (Simoa) technology to show that exosomal α-synuclein oligomer levels were significantly associated with reduced executive function scores (e.g., Stroop test) in PD-MCI patients (34). Mechanistic studies by Chung et al. demonstrated that exosome-mediated α-synuclein propagation triggers microglial inflammatory responses, leading to synaptic loss in the frontal cortex and consequent cognitive impairment (35). Growth-associated protein 43 (GAP43) and synaptosomal-associated protein 25 (SNAP25) are key proteins for synaptic plasticity and neurotransmitter release; changes in their exosomal levels reflect synaptic integrity. Svenningsson et al. reported a 40% decrease in GAP43 content in blood exosomes of PD-MCI patients compared to healthy controls, correlating with hippocampal volume reduction (36). Bacioglu et al. found significantly reduced exosomal SNAP25 in PD-MCI patients, associated with weakened functional connectivity in the default mode network (DMN), indicating decreased synaptic transmission efficiency (37). Chen et al., combining exosomal proteomics with structural MRI, identified that GAP43/SNAP25 levels and frontal cortex thickness together predict the risk of PD-MCI progressing to dementia (38).

Neurofilament Light Chain (NfL) is a cytoskeletal protein released following neuronal axonal damage. Elevated plasma NfL levels reflect the activity of neurodegeneration. Backstrm and colleagues, after a 5-year follow-up, found that initial plasma NfL levels in PD-MCI patients were higher than in PD patients without cognitive impairment, and NfL for each increase of 10pg/mL , the risk of dementia conversion increased 2.3-fold (39). Byrne et al. demonstrated that combining plasma NfL with midbrain atrophy MRI can predict the rate of cognitive decline in PD-MCI patients (40). The rate of white matter atrophy and ventricular enlargement are also considered key early predictive factors for MCI, further indicating that plasma NfL combined with midbrain atrophy MRI has potential in predicting the rate of cognitive decline in PD-MCI patients. Hansson et al. through autopsy research, found that plasma NfL levels positively correlate with the degree of nigral dopaminergic neuron loss and α -synuclein pathological burden in the brains of PD - MCI patients (41).

### 3.3 Saliva/urine

The amount of salivary α-synuclein is positively correlated with its concentration in CSF (cerebrospinal fluid), demonstrating potential screening value. Vivacqua et al. showed that the content of α -synuclein oligomers in the saliva of PD (Parkinson’s disease) patients is significantly positively correlated with its concentration in CSF, and that PD-MCI (Parkinson’s disease with mild cognitive impairment) patients have higher salivary oligomer levels than PD patients without cognitive impairment (42). Stewart et al. used ultrasensitive single molecule detection technology (Simoa) to prove that the total α-synuclein level in saliva highly matches the total α -synuclein concentration in CSF, and that salivary α -synuclein is reduced by approximately 30% in PD-MCI patients compared to healthy controls (43). Kang et al.’s longitudinal cohort study showed that PD patients with high baseline saliva α -synuclein levels are more likely to develop MCI within 2 years, demonstrating its predictive role (44). In PD-MCI patients, oxidative stress participates in cognitive decline by damaging neurons and synaptic function; elevated urinary 8-hydroxy-2′-deoxyguanosine (8-OHdG) levels can serve as a non-invasive pathological marker. A cross-sectional study reported that urinary 8-OHdG levels are approximately 2.5 times higher in PD-MCI patients than in healthy controls, and are significantly negatively correlated with Montreal Cognitive Assessment (MoCA) scores; patients with high 8-OHdG levels have lower executive function and memory scores (45). A five-year cohort study showed that PD patients with baseline urinary 8-OHdG levels above the median have a 2.8-fold increased risk of progression to MCI, and the rate of increase in 8-OHdG levels positively correlates with the rate of cognitive decline (46). At present, there are relatively few studies on saliva/urine biomarkers of PD-MCI. Subsequently, it is necessary to optimize the detection technology, standardize the research process, and deeply explore its value in the early diagnosis of the disease, disease monitoring and mechanism interpretation.

### 3.4 Skin/sweat

α -synuclein abnormal deposition in cutaneous nerve endings and sweat glands is a core pathological feature of PD, and its quantitative detection provides a non-invasive method for early diagnosis of PD-MCI. Donadio et al. found through skin biopsy that the positivity rate of phosphorylated α -synuclein (p- α -syn) deposition in the epidermal small nerve fibers of PD patients reached 90%, and the deposition density in PD-MCI patients was significantly higher than that in PD patients without cognitive impairment. The deposition density was negatively correlated with the MoCA score, indicating its association with cognitive decline (47). Gibbons et al. quantitatively analyzed α -synuclein aggregates around sweat glands using confocal microscopy and found that the aggregate load in PD-MCI patients was three times that of healthy controls and positively correlated with CSF α -synuclein oligomer levels (48). Wang’s team multicenter study showed that skin p-α-syn detection distinguished PD-MCI from healthy controls with an AUC of 0.92, outperforming traditional blood biomarkers such as NfL (49).

## 4 Genetic biomarkers

### 4.1 Genetics

Cohort studies by Sidransky et al. showed that PD patients carrying the heterozygous GBA N370S mutation have a 2.8-fold increased risk of developing MCI compared to non-carriers, with L444P mutation carriers exhibiting an even higher risk (50). Polymeropoulos et al. found that 80% of PD patients carrying the SNCA A53T mutation progressed to MCI within 3 years after diagnosis, significantly higher than non-carriers (51). Kiely et al. reported that the SNCA E46K mutation accelerates α -synuclein fibril formation, which is associated with temporal cortical atrophy and memory impairment (52). Irwin et al. found that PD-MCI patients carrying APOE ε4 exhibit significantly elevated cerebrospinal fluid p-tau181 levels, indicating aggravated tau pathology (53). Zhang et al.’s fMRI study showed that the Val/Val genotype weakens functional connectivity between the DLPFC and the striatum, exacerbating deficits in cognitive flexibility (54).

### 4.2 Epigenetics

Abnormal methylation of genes such as HDAC4 and HOXC5 is present in the peripheral blood of PD-MCI patients and is associated with neuroinflammation and synaptic plasticity. Studies have found hypermethylation in the promoter region of HDAC4 in the peripheral blood of PD-MCI patients, leading to decreased expression, which is also linked to reduced synaptophysin in the frontal cortex. Low expression of HDAC4 impairs synaptic plasticity by inhibiting the BDNF (brain-derived neurotrophic factor) signaling pathway (55). Another study using epigenome-wide association study (EWAS) showed hypomethylation in the gene body of HOXC5 in PD-MCI patients, resulting in increased expression. Overexpression of HOXC5 promotes peripheral monocytes to release IL-6 and TNF- α , exacerbating blood–brain barrier permeability and central inflammation (56). Abnormal expression of miR-124 and miR-132 regulates tau phosphorylation and synaptic function. Relevant studies found a 50% reduction of miR-124 levels in the plasma of PD-MCI patients compared with controls, with a negative correlation between its expression and CSF p-tau181. Experimental evidence shows that overexpression of miR-124 can inhibit GSK-3β activity, thereby reducing tau phosphorylation (57). Additionally, studies have found significantly reduced miR-132 levels in exosomes from PD-MCI patients, with its expression positively correlated with hippocampal volume and memory scores. Overexpression of miR-132 can rescue synaptic plasticity deficits (58).

## 5 Digital biomarkers

Digital biomarkers leverage technologies such as wearable devices, voice analysis, and cognitive task interactions to collect real-time, non-invasive physiological, behavioral, and environmental multidimensional data, greatly enhancing early diagnostic accuracy for PD-MCI. Research shows that digital features based on gait dynamics (such as stride variability) and micro-expression recognition can precisely capture neurodegenerative changes in the prodromal phase of PD-MCI (59). Moreover, smartphone applications (e.g., “PD-Brain”) can diagnose risks of hippocampal and prefrontal cortex functional decline by real-time monitoring of cognitive-motor dual tasks (60). Longitudinal studies indicate that baseline speech articulation deficits in PD patients (e.g., prolonged consonant-vowel transition time) can predict the risk of developing MCI within 2 years, with sensitivity comparable to hippocampal volume reduction (61). These technological advances overcome the subjective limitations of traditional neuropsychological scales, providing objective and dynamic quantitative tools for precise stratification and intervention in PD-MCI.

## 6 Clinical and neuropsychological assessment

Clinical assessment indicators, as a key part of early diagnosis of PD-MCI (Parkinson’s Disease Mild Cognitive Impairment), are typically implemented using standardized clinical evaluation tools. These tools include the Unified Parkinson’s Disease Rating Scale (UPDRS), Montreal Cognitive Assessment (MoCA), and Stroop Color-Word Test, which effectively assess patients’ motor function and cognitive status. Research shows that clinical assessment indicators have significant correlations with imaging and biochemical biomarkers, providing critical information on disease progression. For example, integrating UPDRS scores with imaging results can more accurately diagnose patients’ disease progression and quality of life (62). By combining clinical assessments with other biomarker information, physicians can develop personalized treatment plans better suited to patients’ needs to improve their quality of life.

## 7 Multimodal integration models

The application of machine learning algorithms in the medical field is becoming increasingly widespread, especially in the early prediction of diseases. Common machine learning algorithms include categories such as Support Vector Machine (SVM), Random Forest (RF), Gradient Boosting Tree (GBM), and deep learning models. These algorithms identify biomarkers and potential therapeutic targets of PD-MCI by analyzing different dimensions of the data, and provide new ideas for the comprehensive diagnosis and personalized treatment of PD-MCI. As algorithms continue to be optimized and datasets become increasingly rich, the accuracy and reliability of early machine learning predictions are constantly improving. Machine learning helps identify PD-MCI patients by analyzing specific proteins or molecules in samples such as blood and cerebrospinal fluid. Zhang et al. constructed support vector machine (SVM), decision tree (DT), and logistic regression (LR) models based on multimodal markers of quantitative electroencephalogram (qEEG) and structural magnetic resonance imaging (sMRI). The study found that among 23 pairs of PD-MCI and non-dementia Parkinson’s disease (PD-ND) patients matched by propensity scores, qEEG features are superior to sMRI features in terms of prediction accuracy and area under the curve (AUC). Among them, the theta wave in lead P3 of qEEG features has a significant impact on the classification model, suggesting that multimodal “composite markers” have potential value for individualized diagnosis of PD-MCI (63). Zhu et al. integrated clinical features, plasma markers, functional connectivity of the default mode network and cortical thickness to construct a model and found that the plasma phosphorylated tau181 level and the ratio of phosphorylated tau181/β-amyloid protein 1–42 in the PD-MCI group were significantly higher than those in the PD with normal cognition (PD-NC) group. Moreover, in terms of functional connectivity, the connection between the left posterior cingulate cortex and the left parahippocampal gyrus in the PD-MCI group was enhanced (64). Andrew et al. found that by using support vector machine (SVM) to classify clinical data, T1-weighted magnetic resonance imaging (MRI), resting-state functional MRI (Rs-fMRI), and plasma nerve fiber light chain (NfL) and glial fibrillary acidic protein (GFAP) level data, the results showed that The classifier combining clinical data, Rs-fMRI and NfL biomarkers performed the best, highlighting the importance of multimodal data fusion in improving the predictive ability of PD-MCI (65). A study analyzed the cognitive function assessment of MCI patients using machine learning algorithms. The results showed that a multimodal model integrating multi-source data biomarkers such as neuroimaging, humoral biomarkers, and clinical features demonstrated higher sensitivity and specificity in predicting cognitive decline in patients compared to single biomarkers or traditional diagnostic methods. It has laid a solid foundation for achieving precise and early diagnosis of PD-MCI and promoting the implementation of individualized intervention strategies (66).

## 8 Conclusion

PD-MCI is an intermediate transformation stage from normal cognition to PDD. As the duration of the disease increases, patients gradually lose their autonomy, increasing the mental and economic burden on society and families. To improve the prognosis of patients with PD-MCI, enhance the quality of life and reduce the social burden, the early identification, diagnosis and treatment of PD-MCI are of vital importance. However, there are still difficulties in the early and accurate identification of PD-MCI. This study integrates multi-dimensional data of multimodal biomarkers, including imaging biomarkers, humoral biomarkers, genetic and molecular biomarkers, digital biomarkers, and clinical evaluations. With the help of machine learning algorithms to mine potential disease patterns, it can more comprehensively assess the patient’s condition and improve the sensitivity and specificity of early diagnosis of PD-MCI. Provide quantitative basis for individualized risk stratification.

Although multimodal biomarkers have shown great potential in improving the accuracy, efficiency and personalization of the early diagnosis of PD-MCI, there are still many bottlenecks that need to be urgently broken through in their clinical transformation: (1) The cognitive barriers and trust deficits of clinicians toward emerging technologies stem from the insufficient update of interdisciplinary knowledge; (2) The update of clinical practice guidelines lags behind the progress of scientific research, and there is a lack of standardized procedures for interpreting multimodal data. (3) Insufficient allocation of interdisciplinary research resources has restricted the efficiency of large-scale cohort validation and technology transfer. Multimodal biomarkers still need to be further optimized and validated through clinical tests. Future research directions include: improving data quality on the basis of multimodal data fusion, constructing more generalized integrated models based on neurophysiological and pathological mechanisms, building larger-scale multi-center standardized databases, and promoting clinical transformation and precision diagnosis and treatment through industry-university-research collaboration. By continuously promoting the development of multimodal biomarkers in the diagnosis of PD-MCI, it is expected that in the future, through strengthening interdisciplinary cooperation, such as close collaboration among clinicians, data scientists and biomedical researchers, relying on large-scale cohort validation and standardized processes to promote the clinical application of multimodal biomarkers, more accurate diagnosis and more effective treatment can be provided for PD-MCI patients. In addition, with the continuous advancement of technology, the application of artificial intelligence and machine learning will also play a key role in this process. Utilizing artificial intelligence for multimodal data analysis and pattern recognition can accurately identify disease states and patient needs, providing support for the optimization of treatment plans. In conclusion, although there are many challenges at present, with the deepening of research and technological innovation, the application prospects of multimodal biomarkers in the diagnosis of PD-MCI will be very broad.
